# Paralanguage as a tool for shaping stress response in Listeners: Multimodal physiological sensing study

**DOI:** 10.1016/j.cpnec.2025.100309

**Published:** 2025-06-21

**Authors:** Marina Saskovets, Mykhailo Lohachov, Zilu Liang

**Affiliations:** a Faculty of Engineering, Kyoto University of Advanced Science (KUAS), 18 Yamanouchi Gotanda-cho, Ukyo-ku, Kyoto, 615-8577, Japan; b Institute of Science Tokyo, Department of Civil and Environmental Engineering, M1-14, 2-12-1 Ookayama, Meguro-ku, Tokyo, 152-8552, Japan

## Abstract

**Background:**

Sound is a powerful cue that can influence emotional and physiological states. While musical sounds have been widely studied for their stress-reducing effects, less attention has been given to the role of paralanguage. This study investigates whether a soothing vocal intonation beyond its semantic content can facilitate stress recovery by modulating neurophysiological and biochemical stress markers.

**Methods:**

Thirty-five participants underwent a standardized stress induction task before being exposed to one of three conditions: a soothing voice narration, a robotic voice narration, or silence. Prefrontal cortex (PFC) hemodynamic activity was recorded using functional near-infrared spectroscopy (fNIRS), while stress biomarkers, including salivary cortisol and electrodermal activity (EDA), were measured at multiple time points. The Laterality Index Response (LIR) was computed to assess stress-related cortical asymmetry.

**Results:**

Stress induction significantly increased cortisol levels, EDA, and right-lateralized PFC activation across all groups. During the recovery phase, the soothing voice group demonstrated a significantly faster cortisol reduction compared to both control groups. fNIRS data revealed distinct PFC hemodynamic patterns, with the soothing voice condition shifting activation toward Brodmann areas 45 and 9. However, EDA recovery patterns did not differ significantly across groups.

**Conclusion:**

These findings highlight the potential of paralanguage, specifically soothing voice intonation, in accelerating physiological stress recovery. The observed modulation of cortisol and PFC activity suggests that auditory interventions incorporating emotional prosody could enhance stress regulation strategies. Future research should explore individual differences in response to paralanguage-based interventions and their broader clinical applications.

## Highlights

• Soothing voice intonation enhances cortisol recovery after acute stress.
•FNIRS shows distinct prefrontal activation patterns during stress and recovery.
•Laterality Index Response (LIR) reveals stress-induced cortical asymmetry.
•Findings support the therapeutic potential of paralanguage in stress management.

## 1 Introduction

Sound-based interventions are increasingly being explored as a promising way to promote mental health and well-being. A growing body of research suggests that auditory stimulation, including music, poetry, and natural sounds, can regulate physiological stress responses and support emotional regulation [1,2]. Recent clinical studies have shown that music interventions have positive effects, ranging from improving cognitive functions in Alzheimer's patients to reducing pain during surgery [3,4].

However, it is important to distinguish between music therapy, which typically involves structured, clinician-guided protocols tailored to individual needs, and music exposure, which refers to passive listening without therapeutic intent or professional involvement [5,6]. These approaches may produce different outcomes depending on factors such as listener engagement, context, and delivery format [1]. A comprehensive systematic review and meta-analysis by de Witte et al. demonstrated that music therapy interventions have a medium-to-large effect on reducing stress-related outcomes, highlighting the efficacy of both active and passive music-based approaches in modulating physiological stress responses [7].

Many of these studies focus on *music exposure*, which has demonstrated significant benefits in various medical contexts. For instance, research on patients during cerebral angiography showed that musical support during surgery helps stabilize cortisol levels, blood pressure, and subjective negative experiences such as fear [8]. *Music therapy* has also been shown to reduce anxiety, pain, and morphine consumption during both the operative and postoperative periods [9].

At the same time, an emerging body of research suggests that the effects of music and speech may not be as far apart as previously thought. A meta-analysis by Jansen et al. reveals a significant connection between musical abilities and speech prosody perception, particularly in non-native contexts, emphasizing the shared neural mechanisms underlying pitch processing for both music and language [10]. Further supporting this connection, Maess et al. demonstrated that Broca's area and its right-hemisphere counterpart play a key role in processing musical syntax, pointing to overlapping neural pathways that govern rule-based information processing across both language and music [11]. This convergence suggests that the therapeutic impact of auditory stimuli may arise from domain-general cognitive and affective mechanisms, rather than being limited to either music or speech alone.

Speech-based auditory stimuli, such as poetry and expressive narration, have also been shown to reduce stress and anxiety. Studies by Jabarouti et al. demonstrate that classical Persian poetry significantly reduces stress hormone levels, emphasizing the therapeutic potential of its rhythmic structure [12]. Mohammadidan et al. reported that poetry therapy can effectively reduce depression, anxiety, and stress levels, emphasizing the role of expressive and culturally resonant auditory stimuli in facilitating emotional regulation [13]. Stein Duker et al. demonstrated that listening to audiobooks effectively reduces fear and state anxiety in children, emphasizing the potential of auditory distractions in modulating emotional responses during stressful situations [14]. Moreover, a systematic review and meta-analysis demonstrate that exposure to natural sounds can reduce physiological stress markers, reinforcing the restorative potential of auditory stimuli in stress management [15].

Furthermore, the recent popularity of Autonomous Sensory Meridian Response (ASMR) also underscores the potential of voice-based stimuli in emotional regulation. ASMR often features human whispering and soft vocalizations that elicit relaxation. Although empirical research on ASMR is still emerging, initial studies suggest it may reduce heart rate and increase skin conductance, a complex pattern of autonomic activation. Poerio et al.'s findings suggest that ASMR elicits both calming and arousing physiological responses, reflecting the nuanced nature of autonomic regulation during such experiences [16].

### 1.1 Neurobiology of sound intervention

Despite the promising outcomes, the mechanism through which sound therapy exerts its effects is not well understood. In current studies, music or poetry is often regarded as a holistic phenomenon, rooted in emotional and cultural significance.

Evidence from neuroscience indicates that auditory stimuli can modulate stress responses through subcortical-limbic circuits, involving the amygdala and hippocampus, which are integral to the regulation of the hypothalamic-pituitary-adrenal (HPA) axis. The amygdala processes emotionally charged or threatening stimuli and acts as a stress response amplifier. It sends excitatory projections to the hypothalamus, activating the HPA axis, leading to cortisol release [17]. Simultaneously, the hippocampus contributes to the contextual evaluation of stressors and exerts inhibitory control over the HPA axis, promoting the negative feedback control of cortisol secretion and contributing to the termination of the stress response [18].

There is growing evidence of the close interconnection between auditory processing and the limbic system activity. Kraus et al. discuss how stressful acoustic stimuli, such as noise, can cause amygdala-mediated release of stress hormones [19]. On the other hand, Koelsch in his paper “Brain correlates of music - evoked emotions” discusses how music listening affects the hippocampal formation and modulates stress responses by engaging hippocampal pathways [20]. Additionally, research by Derner et al. have shown that auditory stimulation can influence hippocampal rhythms, with implications for enhancing memory and mitigating cognitive decline [21]. Furthermore, Reybrouck et al. have demonstrated that music listening recruits large-scale brain networks, including the hippocampus, involved with emotion and memory processing [22].

A more detailed understanding of how specific acoustic features influence neural and hormonal stress systems could refine therapeutic interventions, tailoring sound therapies to individual needs and clinical conditions, and broadening their application in healthcare.

### 1.2 Objective and hypothesis

We aim to explore the therapeutic potential of soothing human voice. Our research exclusively focuses the paralinguistic component of human speech, which refers to the acoustics independent of semantic content. Our research investigates how these non-verbal vocal elements affect physiological stress, a universal systemic response associated with a wide range of physical and mental health conditions. Stress is well known for its integrative effects: it involves the whole body through sympathetic activation and changing biochemical balance [23]. Building on this physiological perspective, we focus specifically on objective, quantifiable stress markers, including salivary cortisol, electrodermal activity (EDA), and functional near-infrared spectroscopy (fNIRS). These markers offer complementary insights into stress physiology. Cortisol captures slower hormonal dynamics, EDA reflects rapid autonomic arousal, and fNIRS-based hemodynamics reveal cortical mechanisms of stress processing. While partially overlapping, these systems are not fully redundant and allow for a multimodal view of how paralanguage influences stress recovery [24].

Previous studies have shown that both electrodermal activity and prefrontal cortex hemodynamics are sensitive to auditory stimulation and reliably reflect autonomic and cortical dynamics associated with stress regulation. fNIRS demonstrated its reliability for estimating stress-related brain activity, particularly in the prefrontal cortex [25]. Previous research has also demonstrated that fNIRS can be effectively used to assess brain responses during sound-based interventions. For instance, exposure to white noise has been found to reduce cortical excitability in the prefrontal cortex, indicating a sedative effect that may contribute to stress reduction [26]. Similarly, binaural beats stimulation has been associated with the mitigation of mental stress, as evidenced by changes in prefrontal cortex activity measured through fNIRS [27]. Additionally, EDA measurements have been used to assess the physiological stress response induced by various stimuli, confirming its role as a reliable indicator of autonomic nervous system activity [28].

The originality of this work lies in its exclusive focus on the therapeutic effect of the non-verbal components of human speech. We compare exposure to a soothing human voice with two control conditions: a monotonic robotic voice and silence. This study builds upon a theoretical framework developed through our prior scoping review, which synthesized evidence on the mechanisms by which auditory stimuli may influence stress regulation [1,29] and provides experimental evidence to assess the role of paralanguage in stress regulation.

**Study objective:** evaluate prefrontal cortex (PFC) activity, sympathetic markers (EDA) and biochemical stress markers (cortisol) during soothing voice intervention after stress task in healthy subjects.

Study hypothesis:

1) Listening to a soothing human voice will lead to a faster reduction in cortisol and EDA levels compared to a robotic voice or silence.
2) Hemodynamic signals in the prefrontal cortex will reflect greater recovery (e.g., normalization of frontal asymmetry, faster decrease of the oxyhemoglobin concentrations) in the soothing voice condition.

## 2 Methods

### 2.1 Participants

Participants were recruited by distributing flyers around the Kyoto University of Advanced Science campus. Interested individuals completed an online screening form assessing eligibility. Inclusion criteria were as follows: age between 18 and 35 years, normal hearing, no self-reported history of neurological or psychiatric disorders, and English language proficiency at an upper-intermediate level or higher. Japanese language proficiency was also assessed using self-reported comprehension levels. Participants with elementary Japanese skills or lower were eligible for the sound intervention groups, while those with higher Japanese fluency were assigned to the passive control group (silence condition). This allocation ensured that participants in the intervention groups were unlikely to understand the story content, isolating the effect of vocal acoustics from semantic processing. Neither English nor Japanese was the native language for the majority of participants. Most of the volunteers were international students residing in Japan. The age criterion was selected as optimal for fNIRS measurements in adults, as pilot tests indicated the device's optimal signal quality in younger adults.

Exclusion criteria were chronic medical conditions, use of hormonal or psychotropic medications, smoking, recent major life stressors, and substance abuse within the previous month. These were assessed through a self-report questionnaire administered at the initial screening. Menstrual cycle phase was not assessed in this study, which we acknowledge as a limitation given their potential influence on cortisol levels.

Out of 44 individuals who responded to the recruitment call, seven participants withdrew before completing the experiment: due to health issues (N = 1), ineligibility discovered during the pre-test interview (N = 2), or scheduling conflicts (N = 4). After preliminary screening, 37 met the eligibility criteria and were invited to participate. Two participants were excluded from the analysis due to poor fNIRS signal quality or technical interruptions. The final sample included 35 participants (mean age = 23.0 years, SD = 5.0; 16 female, 19 male), who were randomly assigned to one of three experimental conditions: soothing voice condition (n = 12), robotic voice condition (n = 11), and silence condition (n = 12). There were no significant group differences in age or gender distribution. Most participants were non-native English speakers, with 28 reporting advanced proficiency, 4 reporting upper-intermediate proficiency, and only 3 identifying as native English speakers. Only 1 participant was a native Japanese speaker, minimizing potential linguistic or cultural confounds.

Eligible participants were then scheduled for a meeting and provided with a written and oral description of the study procedures. To control for factors that may influence stress biomarkers, they were instructed to avoid alcohol for 48 h before the experiment, refrain from coffee and physical exercises on the experiment day and ensure adequate sleep the night before. As compensation, participants received a reward equivalent to 20 USD for their involvement in the study.

## 3 Ethics statement

This study was conducted following the Declaration of Helsinki. The study protocol has been approved by the Ethics Review Board of the Kyoto University of Advanced Science. All participants have given their written consent.

### 3.1 Study design

The study employed a between-subjects experimental design to evaluate the effect of human speech acoustics (independent variable) on physiological markers of stress, including salivary cortisol, sympathetic arousal reflected through electrodermal activity, and prefrontal cortex hemodynamic measured by functional near-infrared spectroscopy (dependent variables). In our study, we separated acoustic properties from semantic content by presenting a speech in Japanese, a foreign language to the participants in the intervention groups.

Three acoustic conditions were used, with each condition assigned to a different group of participants after baseline recording and stress induction: (1) soothing voice condition (intervention group): participants listened to the Momotaro folk tale record, narrated in Japanese by a professional storyteller, delivered in a warm and soothing tone; (2) robotic voice condition (active control): the same Japanese story was presented using a flat, synthetic voice generated by a basic text-to-speech program with no emotional modulation; and (3) silence condition (passive control): participants rested quietly wearing sound-muffling headphones, with no auditory input.

Participants were randomly assigned to one of the three conditions, but those with a Japanese level higher than elementary were assigned to the silence group to prevent the content of the speech from influencing the acoustic effects.

In addition to self-report, language comprehension was verified using a brief translation task from an unrelated folktale: those unable to translate even basic words (e.g., “mountain,” “river,” or “man”) were considered non-comprehending and were assigned to one of the two sound conditions. Four people, who were able to interpret the general meaning of the passage were reassigned to the silence group along with the single native specker.

Participants were informed about the general procedures but were blinded to the specific study hypotheses. The final groups included 12 participants in the soothing voice condition, 11 in the robotic voice condition, and 12 in the silence condition. All experiment sessions were conducted between 2:00 p.m. and 4:00 p.m. to minimize the confounding effect of cortisol's circadian rhythm.

**Fig. 1:**
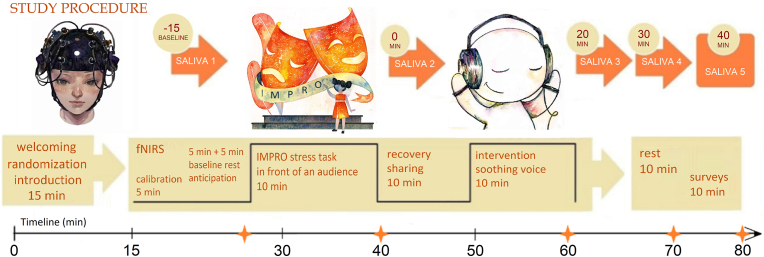
Study design.

The procedure was designed to provoke stress by combining social evaluation with cognitive demands, consistent with established principles used in validated protocols such as the Trier Social Stress Test (TSST). Its effectiveness in triggering physiological responses, including cortisol secretion, electrodermal activity, and prefrontal cortex dynamics, was evaluated within the current experimental setting and is detailed in our complementary publication [30]. A schematic overview of the study procedure is presented in (Fig. 1).

### 3.2 Stress induction

Stress was induced using an improvisation speaking task, which consisted of three phases with increasing difficulty levels. The process began with a preliminary preparation phase, during which monitoring devices were placed to record brain and body signals. This was followed by a 5-min rest, where participants sat quietly to establish baseline measurements. Next, participants underwent a 5-min anticipation stage, during which they received detailed instructions about the upcoming tasks. During this phase, participants were allowed to ask clarifying questions or mentally prepare in silence before the stress induction that followed.

The stress induction phase lasted 10 min and was divided into three progressively challenging stages: free improvisation, a random word challenge, and an arithmetic load. Two listeners were present throughout the 10 min, and video recordings were made for further analysis. The protocol was framed as a game, with each stage introducing more complexity.

**Task A (3 min):** Participants were asked to speak continuously on any topic without pausing for more than 5 s. If a pause exceeded 5 s, the task would restart. This stage served as a warm-up, allowing participants to establish a performance strategy.

**Task B (4 min):** Participants continued speaking while incorporating random words introduced aloud by the listeners. They were required to integrate these words into their narrative without pausing. This task encouraged verbal creativity, with no right or wrong approach. Researchers noted the participants’ strategies, such as repetition, integration, or ignoring the words.

**Task C (3 min):** Participants maintained continuous speech while solving arithmetic problems presented aloud by the listeners. They had to provide answers immediately while continuing their narrative. In this task, participants are supposed to be distracted from their main activity by questions from different domains (interruption of the narrative activity with calculations). The level of cognitive stress increased even more while participants were expected to give a single correct answer for each problem. At the same time, listeners did not comment on accuracy or stop the game in the case of mistakes. Throughout all tasks, listeners refrained from non-verbal support (e.g., smiling or nodding) to enhance stress.

After completing the three tasks, participants entered a 5-min intermediate rest period, seated comfortably in a chair. During this time, they engaged in a brief, informal conversation with the lead researcher, who conducted a semi-structured interview about their subjective experience with questions like, “How did you feel?” and “What was your experience during the task?".

### 3.3 Sound intervention

In our study, we aimed to isolate the paralinguistic components of human speech. To eliminate the influence of semantic content, we used the same text — a classical folk tale “Momotaro” — for both the intervention group and the control group 1. However, in the control group 1, the intonation and delivery were mechanically altered to a robotic, emotionless voice. This comparison allowed us to isolate the effect of speech prosody while controlling for potential confounding variables, such as background noise or other acoustic stimulation unrelated to paralanguage.

As this type of intervention is relatively new, there is limited literature to guide the selection of a specific voice sample. We chose Japanese for practical reasons related to participant recruitment. Many students at Kyoto University of Advanced Science are newcomers who have not yet learned the language. At the same time, they are exposed to the linguistic environment and are already accustomed to the sound of Japanese.

Therefore, we consider this language as a familiar stimulus that would help mitigate the confounding effects of an orienting response [31].

In addition, we opted for a single standardized track delivered via headphones and rejected the option of a live performance or the use of multiple audio records. This decision was made to avoid the potential influence of personal preferences or interpersonal engagement that could arise from different voices or varied delivery styles.

After the sound intervention, participants rested for 10 min before providing their fourth saliva sample. The monitoring devices were then removed. During the debriefing stage, the researcher provided feedback and answered any questions participants had about the study. Participants were also invited to share their thoughts on the experiment, including the study design, the comfort and functionality of devices, their interactions with the listeners, and their overall experience. This discussion followed a free-narrative approach, allowing participants to express their opinions openly. The main discussion lasted for 10 min, then the final saliva sample was collected.

## 4 Measurements

To investigate the effect of human voice acoustics on stress levels, we analyzed multiple physiological and neural markers of stress. These included cortisol levels as a reliable indicator of stress, EDA to assess sympathetic arousal, and brain responses through hemodynamic changes in the prefrontal cortex measured with a fNIRS device. This approach aimed to capture stress-related changes and recovery dynamics using objectively measurable physiological data.

### 4.1 Cortisol sampling

Salivary cortisol, a widely used biomarker of HPA axis activity, was measured using the SOMA CUBE system (SOMA Bioscience, Oxford, UK), which employs lateral flow immunochromatographic strip (ICS) technology, which has demonstrated results comparable to ELISA-based laboratory assays [32]. Saliva samples were collected using passive drool into sterile polypropylene tubes at five predefined time points during the experimental session: (1) “baseline,” following an initial resting period, before the onset of the stress task; (2) “stress,” immediately following the stress induction task; (3) “sound,” right after the experimental sound intervention (e.g., listening to the “Momotaro” folktale or resting in silence); (4) “rest-1,” 10 min after the sound intervention; and (5) “rest-2,” 20 min after the sound intervention. The SOMA CUBE analyzer provided output in nanomolar (nM) concentrations. For interpretation, 1 nM (nM) is equivalent to 1 nmol/L, the standard reporting unit for salivary cortisol.

### 4.2 Electrodermal activity (EDA)

Electrodermal activity, a measure of sympathetic nervous system activation, was continuously recorded using the Empatica E4 wristband (Empatica Inc., Cambridge, US) wristband, an FDA-approved research-grade device. The E4 uses two dry silver-plated electrodes placed on the inner surface of the wrist to measure skin conductance through non-invasive contact with the skin. In this study, two wristbands were worn on the both hands to ensure signal redundancy, reduce the impact of motion artifacts, and allow cross-validation of physiological signals. Data were sampled at 4 Hz and recorded in microsiemens (μS).

### 4.3 Prefrontal cortex hemodynamics

Hemodynamic responses in the prefrontal cortex were recorded using a wearable Artinis Brite 24 functional near-infrared spectroscopy system, sampling at 50 Hz. fNIRS is a non-invasive, portable neuroimaging method that estimates brain activity by measuring changes in oxygenated (OHb) and deoxygenated (HHb) haemoglobin in the cortical surface. HbO reflects the concentration of haemoglobin bound with oxygen and typically increases with neural activation due to greater cerebral blood flow, while HHb refers to haemoglobin not bound to oxygen. The abbreviation HHb, though seemingly redundant, is a standard term in fNIRS research used to denote deoxygenated haemoglobin due to historical reasons. Compared to fMRI, fNIRS offers lower spatial resolution but has important advantages in terms of cost, portability, and tolerance to motion, making it especially suitable for naturalistic and mobile experimental designs like ours.

The system consisted of 24 optodes arranged in a 2 × 12-channel configuration, fixed on a soft neoprene head cap. The optodes were symmetrically distributed over the right and left sides of the frontal area, with half positioned between the FpZ-F7-F3-FZ region and the other half between the FpZ-F8-F4-FZ region, following the international 10–20 EEG system. This configuration targeted the frontopolar and dorsolateral regions of the PFC, approximately corresponding to Brodmann areas 9, 10, 45, 46, 47, and allowed for the measurement of frontal asymmetry, an important marker of mental stress. Data were streamed in real-time via Bluetooth into OxySoft (Artinis Medical Systems), and subsequently preprocessed and analyzed using custom Python 3.10 scripts. The wearable design of the device facilitated participant mobility, ensuring ecological validity by simulating naturalistic conditions. These design and measurement protocols align with prior research on brain activity under stress-inducing conditions [30].

### 4.4 Data analysis: signal preprocessing

**EDA** signals were exported from the Empatica E4 wristband and exported as CSV files for preprocessing. The raw data occasionally contained gaps, which were caused by random sensor detachment during the experiment sessions. These detachment events, lasting up to a few seconds, were not correctable using standard filtering techniques. To address this, a novel error detection algorithm was developed. This algorithm identified measurement errors by detecting abrupt changes in EDA values exceeding 0.01 μS, which fell outside an acceptable range. When errors were detected, the algorithm linearly adjusted the range based on valid neighbouring observations, restoring the EDA signal to the allowable range. Missing data were then filled using linear interpolation. High-frequency noise was further removed, and the EDA signal was smoothed using a low-pass Butterworth filter set at a 0.5 Hz cutoff frequency [33]. After filtering, all participant data were normalized via min-max scaling to fit within a 0–1 range [34].

We focused on tonic-level EDA, also referred to as skin conductance level (SCL), which reflects slow, continuous changes in arousal over time. This parameter was selected due to its robustness in capturing sustained stress responses during longer experimental phases. To synchronize EDA data with task phases, participants used the timestamp button on the device to mark key experimental events. EDA data were then segmented into relevant periods (baseline, stress task, sound intervention, rest) for comparison.

**The fNIRS** signals were exported from OxySoft (version 3) in European Data Format (EDF) for analysis. Relative changes in oxygenated haemoglobin (ΔOHb) and deoxygenated haemoglobin (ΔHHb) concentrations were calculated using the Beer-Lambert law [35], with age-adjusted differential path lengths [36]. To ensure data quality, channels with poor signals were identified and removed using the Scalp Coupling Index (SCI) method [37]. SCI quantifies the quality of optode contact with the scalp by measuring the strength of physiological pulsations, such as cardiac signals, in the fNIRS data [38]. The signal quality assessment began by applying a band-pass filter (0.7–1.5 Hz) to isolate heartbeat components. Then, signals were normalized to even out their amplitudes, and the zero-lag cross-correlation was computed to calculate the SCI, representing the signal-to-noise ratio. Channels with an SCI value below 0.75 were excluded from further analysis [37]. For the remaining channels, a second band-pass filter (0.02–0.18 Hz) was applied to remove high-frequency components, including cardiac and respiratory noise.

Time stamps for EDA and fNIRS measurements were synchronized across experimental stages, and the corresponding ΔOHb, ΔHHb, and EDA values were averaged for each stage to examine group mean differences. A 2-min rolling window was applied to compute these averages, providing an optimal balance between signal smoothing and temporal resolution. This approach minimized transient fluctuations while preserving meaningful physiological trends.

### 4.5 Data analysis: statistical tests

Data analysis was performed using the Pandas and SciPy libraries in Python 3.10. Before the main statistical analysis, the homogeneity of variance was assessed using Levene's test. Analysis of variance (including paired t-tests and ANOVA for repeated measures) was conducted to investigate the effects of the experimental stage, conditions (groups), and their interaction.

### 4.6 Laterality index response (LIR)

The Laterality Index Response (LIR) was calculated to evaluate the relative hemispheric activity in the prefrontal cortex during the experimental conditions [39]. The LIR was defined in Equation (1)

(1) $$LIR=\frac{Right-ROI\;activity-Left-ROI\;activity}{Right-ROI\;activity+Left-ROI\;activity}$$

where ROI represents the region of interest corresponding to specific ΔOHb channel pairs. This index ranges from −1 to +1, with positive values indicating greater activity in the right prefrontal cortex and negative values indicating greater activity in the left prefrontal cortex. LIR was computed for all relevant channel pairs across the experimental stages, including baseline, stress, and sound interventions. Statistical analyses of LIR were performed to assess changes across conditions, employing within-subjects paired t-tests for stress analysis and between-subject t-tests for comparison across groups.

## 5 Results

### 5.1 Cortisol

All participants exhibited a significant increase in salivary cortisol levels in response to the improvisation stress protocol. The repeated-measures ANOVA (3 groups × 2 stages: “baseline” and “stress”) shows significant changes between the baseline (time point = −15 min) and stress (time point = 0 min) across all groups, indicating a main within-subject effect of the “stage” (F (1, 34) = 11.839; p = 0.002; η^2^ = 0.270). However, there were no significant between-subjects effects for the “group” factor, nor were there significant interactions between stage and group during the baseline and stress periods.

**Fig. 2:**
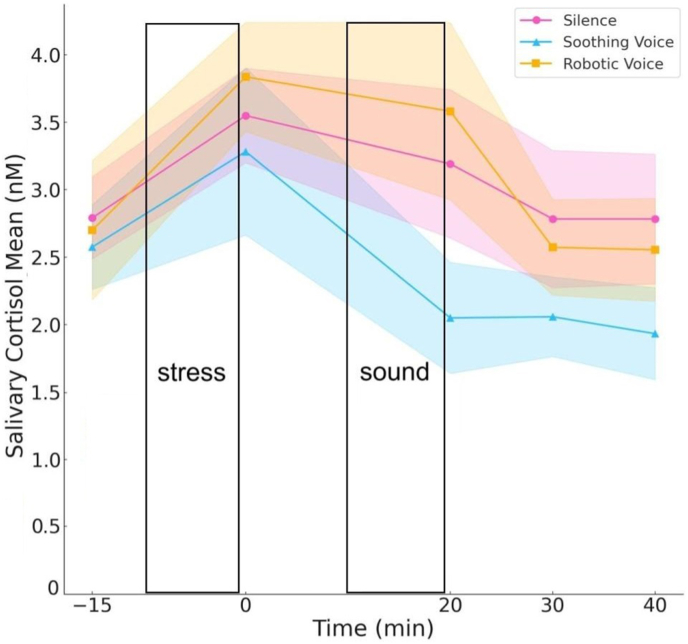
Salivary cortisol levels in response to the stress task and sound intervention.

During the recovery phase, the intervention group demonstrated a notably faster decline in cortisol levels compared to both control groups. The repeated-measures ANOVA (3 groups × 3 stages: “baseline”, “stress”, “sound”) showed significant differences in cortisol levels over time (within-subject effect of the “stage”, F (2,34) = 5.398; p = 0.027; η^2^ = 0.144), and among groups (between-subjects effect “group”: F (2,34) = 17.663; p < 0.001; η^2^ = 0.356). However, the group-by-time interaction was not significant. By the 20-min mark, cortisol levels in the intervention group had returned to baseline, while the control group showed a more gradual decrease, only approaching baseline after the 30-min mark (Fig. 2).

### 5.2 EDA

A two-way repeated-measures ANOVA (3 groups × 5 stages: “baseline”, “stress”, “sound”, “rest-1”, and “rest-2”) was conducted to examine tonic-level EDA responses. The improvisation speaking task induced a noticeable increase in EDA across all groups. The main effect of the stage was significant (F (4, 34) = 94,15; p < 0.001; η^2^ = 0.631), demonstrating reliable sympathetic arousal during the stress phase. However, no significant between-group differences were observed during this phase.

**Fig. 3:**
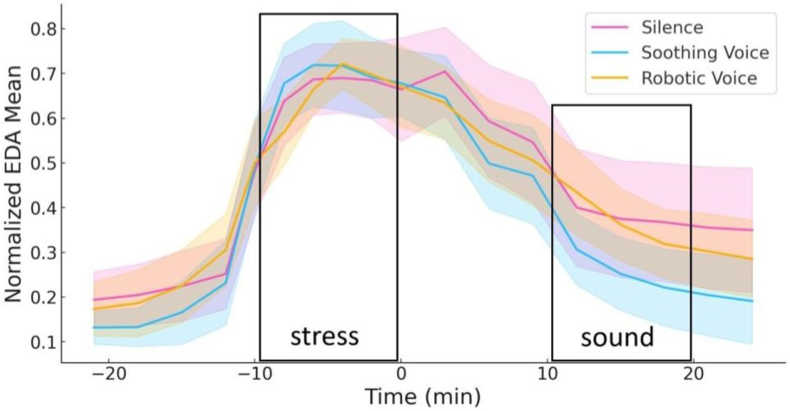
EDA levels in response to the Stress Task and Sound Intervention.

During the recovery period, EDA levels decreased for all groups. Post hoc comparisons revealed no significant differences in recovery rates, suggesting similar autonomic recovery patterns across all conditions (Fig. 3).

### 5.3 fNIRS

**Fig. 4:**
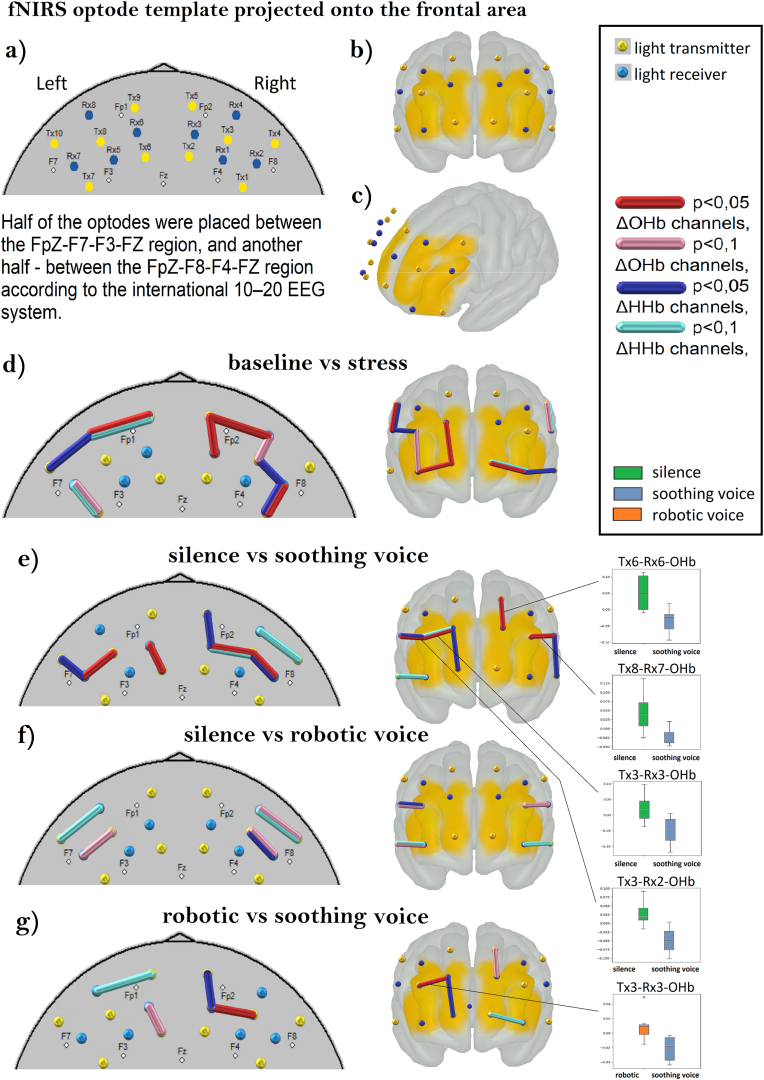
PFC Hemodynamic Response to the Stress Task and Sound Intervention: a) 2D topographic view with the location of transmitters and receivers; b) anterior view; c) left front dorsal view; d) baseline vs stress: within-subjects *t*-test shows a significant increase in hemodynamics during the stress stage compared to the baseline in a set of channels; e) silence vs soothing voice: between-subjects *t*-test for the sound stage; f) silence vs robotic voice: between-subjects *t*-test for the sound stage; g) robotic vs soothing voice: between-subjects *t*-test for the sound stage.

The fNIRS data revealed significant changes in prefrontal cortex hemodynamic activity across different stages (baseline, stress, sound intervention) and between groups. The analysis primarily focused on changes in oxygenated haemoglobin levels, which serve as a marker for cortical activity. Previous research has demonstrated that ΔOHb is a more sensitive indicator of cortical activity than ΔHHb since it refers to the local neural activity, whereas ΔHHb reflects both venous oxygenation and blood volume, complicating its use as a straightforward indicator of cerebral blood flow or oxygen consumption [40]. Paired t-tests were conducted to examine within- and between-group differences (Fig. 4).

#### 5.3.1 fINRS results for stress induction

Paired t-tests revealed a significant increase in ΔO2Hb levels in the prefrontal cortex during the stress induction phase (improvisation speech) compared to the baseline across all groups. Channels Tx1-Rx2, Tx5-Rx3, and Tx5-Rx4 showed significant increases in ΔO2Hb (p < 0.05) in the right hemisphere, with effect sizes ranging from 0.038 to 0.143, indicating strong prefrontal activation with right lateralization (Brodmann areas 10 and 46). At the same time, channel Tx9-Rx8 showed a significant increase in the oxygenated blood flow in the left hemisphere (Table 1).

**Table 1:** PFC hemodynamic response to the stress task.

haemoglobin	channel	t-value	p-value	Cohen's d	hemisphere	Brodmann area
**baseline vs stress: within-subjects *t*-test (p < 0.05)**						
ΔOHb	Tx1-Rx2	−2.737	0.041	−0.038	Right	46
	Tx5-Rx3	−2.763	0.014	−0.121	Right	10
	Tx5-Rx4	−2.327	0.034	−0.143	Right	10
	Tx9-Rx8	−2.389	0.031	−0.051	Left	10
ΔHHb	Tx1-Rx2	8.403	0.0004	0.053	Right	46
	Tx3-Rx2	2.621	0.027	0.045	Right	45
	Tx10-Rx8	2.545	0.022	0.033	Left	47

#### 5.3.2 fINRS results for sound intervention

During sound intervention, participants exhibited significant between-group differences in ΔO2Hb levels. The analysis of the sound stage revealed significant differences in prefrontal activation across groups, as assessed by between-subjects t-tests (p < 0.05). When comparing the silence control, the intervention group showed significantly lower ΔOHb in the right hemisphere at Brodmann areas 45 (Tx3-Rx2, t = 2.281, p = 0.049, Cohen's d = 2.109) and 9 (Tx3-Rx3, t = 2.893, p = 0.012, Cohen's d = 1.704), as well as in the left hemisphere at Brodmann areas 45 (Tx8-Rx7, t = 2.845, p = 0.014, Cohen's d = 1.648) and 9 (Tx6-Rx6, t = 2.682, p = 0.025, Cohen's d = 1.791).

Corresponding ΔHHb changes were also significant in the right hemisphere at Brodmann areas 45 (Tx3-Rx2, t = 2.711, p = 0.026, Cohen's d = 2.917) and 10 (Tx5-Rx3, t = 2.368, p = 0.031, Cohen's d = 1.234), as well as in the left hemisphere at Brodmann area 44 (Tx10-Rx7, t = 2.395, p = 0.047, Cohen's d = 1.856).

Comparisons between the intervention condition and the robotic voice condition revealed significant differences in ΔOHb in the right hemisphere at Brodmann area 9 (Tx3-Rx3, t = 2.451, p = 0.039, Cohen's d = 1.733) and in ΔHHb at Brodmann area 10 (Tx5-Rx3, t = 3.016, p = 0.011, Cohen's d = 1.741).

Furthermore, comparisons between the two control conditions revealed significant differences in ΔHHb in the right hemisphere at Brodmann area 45 (Tx3-Rx2, t = 2.414, p = 0.032, Cohen's d = 1.421) (Table 2).

**Table 2:** PFC hemodynamic response to the sound intervention.

haemoglobin	channel	t-value	p-value	Cohen's d	hemisphere	Brodmann area
**silence vs soothing voice: between-subjects *t*-test for the sound stage (p < 0.05)**						
ΔOHb	Tx3-Rx2	2.281	0.049	2.109	Right	45
	Tx3-Rx3	2.893	0.012	1.704	Right	9
	Tx8-Rx7	2.845	0.014	1.648	Left	45 (Broca)
	Tx6-Rx6	2.682	0.025	1.791	Left	9
ΔHHb	Tx3-Rx2	2.711	0.026	2.917	Right	45
	Tx5-Rx3	2.368	0.031	1.234	Right	10
	Tx10-Rx7	2.395	0.047	1.856	Left	44 (Broca)
**robotic vs soothing voice: between-subjects *t*-test for the sound stage (p < 0.05)**						
ΔOHb	Tx3-Rx3	2.451	0.039	1.733	Right	9
ΔHHb	Tx5-Rx3	3.016	0.011	1.741	Right	10
**silence vs robotic voice: between-subjects *t*-test for the sound stage (p < 0.05)**						
ΔHHb	Tx3-Rx2	2.414	0.032	1.421	Right	45

#### 5.3.3 fINRS results for laterality index response

The analysis of the laterality index response revealed significant differences across experimental conditions, during the stress and sound stages. For the stress stage, within-subjects *t*-test indicated substantial right lateralization from baseline to stress for the channel pairs [Tx9-Rx8, Tx5-Rx4] (t = −6.425, p = 0.023, Cohen's d = −1.381). A similar trend was observed for channel pairs [Tx9-Rx6, Tx5-Rx3], though these did not reach statistical significance (t = −2.355, p = 0.078, Cohen's d = −0.183). Both channel pairs correspond to the orbitofrontal cortex (Brodmann's area 10).

**Fig. 5:**
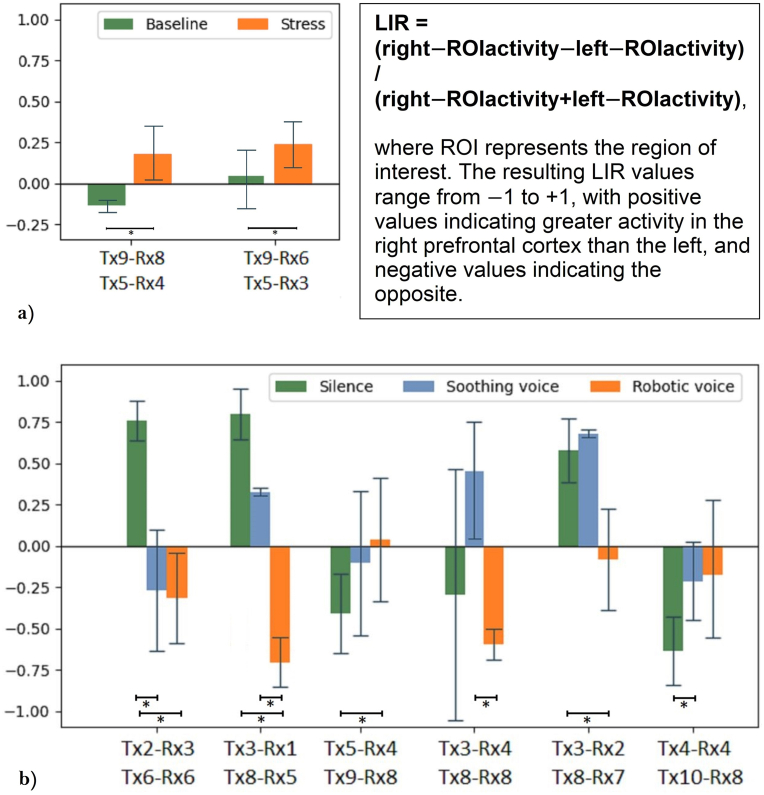
Laterality Index Response to the Stress Task and Sound Intervention: a) stress compared to baseline: within-subject *t*-test; b) soothing voice compared to silence and robotic voice: between-subject *t*-test.

During the sound stage, comparisons across silence, soothing, and robotic voice groups revealed significant effects. Soothing versus robotic voice comparisons revealed significant right lateralization for channel pairs [Tx3-Rx1, Tx8-Rx5]: (t = 10.738, p < 0.001, Cohen's d = 10.679) and [Tx3-Rx2, Tx8-Rx7]: (t = 2.896, p = 0.027, Cohen's d = 2.404)**.** When compared to the silence condition, a soothing voice resulted in left lateralization for channel pairs [Tx2-Rx3, Tx6-Rx6]: (t = −4.642, p = 0.003, Cohen's d = −3.790) and [Tx4-Rx4, Tx10-Rx8]: (t = 2.549, p = 0.034, Cohen's d = 1.981). Furthermore, comparisons between the silence versus robotic voice showed significant differences for channel pairs [Tx2-Rx3, Tx6-Rx6]: (t = 5.489, p = 0.005, Cohen's d = 6.293), [Tx3-Rx1, Tx8-Rx5]: (t = 10.738, p < 0.001, Cohen's d = 10.679), [Tx5-Rx4, Tx9-Rx8]: (t = −2.797, p = 0.013, Cohen's d = −1.475) and [Tx3-Rx2, Tx8-Rx7]: (t = 2.896, p = 0.027, Cohen's d = 2.404). These results suggest that the most significant differences were observed between the two control conditions, revealing a contradictory view on the PFC laterality index sensitivity to soothing voice effects on prefrontal asymmetry (see Fig. 5) (see Table 3).

**Table 3:** Laterality index response to the stress task and sound intervention.

Results for the stress stage	Results for the sound stage
(baseline vs stress: within-subjects *t*-test (∗p < 0.05))	(soothing voice compared to silence & robotic voice: within-subjects *t*-test (∗p < 0.05))
[Tx9-Rx8, Tx5-Rx4] (t = −6.425, p = 0.023, Cohen's d = −1.381) [Tx9-Rx6, Tx5-Rx3] (t = −2.355, p = 0.078, Cohen's d = −0.183)	**silence vs. robotic voice:** [Tx2-Rx3, Tx6-Rx6]: (t = 5.489, p = 0.005, Cohen's d = 6.293) [Tx3-Rx1, Tx8-Rx5]: (t = 10.738, p < 0.001, Cohen's d = 10.679) [Tx9-Rx8, Tx5-Rx4]: (t = −2.797, p = 0.013, Cohen's d = −1.475) [Tx3-Rx2, Tx8-Rx7]: (t = 2.896, p = 0.027, Cohen's d = 2.404) **soothing voice vs. silence:** [Tx2-Rx3, Tx6-Rx6]: (t = −4.642, p = 0.003, Cohen's d = −3.790) [Tx4-Rx4, Tx10-Rx8]: (t = 2.549, p = 0.034, Cohen's d = 1.981) **soothing voice vs. robotic:** [Tx3-Rx1, Tx8-Rx5]: (t = 6.181, p = 0.003, Cohen's d = 7.571) [Tx3-Rx4, Tx8-Rx8]: (t = 3.563, p = 0.023, Cohen's d = 3.563)

## 6 Discussion

The results support the study's hypothesis and underscore the role of paralanguage, in mitigating physiological stress responses. Specifically, listening to a soothing human voice led to a significantly faster reduction in cortisol levels compared to both robotic voice and silence conditions. At the same time, the hypothesis regarding electrodermal activity was not fully supported, as no significant differences were observed in EDA recovery rates across the conditions. FNIRS data revealed significant hemodynamic changes in the prefrontal cortex during stress, and between-group differences during sound intervention, reflecting recovery dynamics on the cortical level.

### 6.1 Cortisol levels

Our findings indicate that participants exposed to the soothing voice intervention exhibited significantly faster cortisol reductions during the recovery phase compared to the two control conditions (i.e., both robotic voice and silence). This aligns with previous research suggesting that auditory stimuli, particularly those incorporating soothing emotional prosody and even rhythmic patterns, effectively mitigate physiological stress response [41]. The rapid return of cortisol to baseline levels in the soothing voice group serves as a ground truth for further measurements.

When comparing effect size and mean differences, it is evident that the stress induction phase had a more pronounced effect than the recovery phase. This difference could indicate that stress responses are typically more immediate and require less time for resolution, while recovery, particularly in response to passive interventions like auditory stimuli, may take longer to manifest. Furthermore, it is important to note the difference in the nature of the interventions; the stress induction protocol involved multimodal, behaviourally engaging tasks, while the recovery phase was a passive auditory intervention without additional sensory or cognitive engagement (e.g., speech content or visual support).

### 6.2 Prefrontal cortex hemodynamics

The fNIRS data revealed significant differences in prefrontal cortex hemodynamics associated with the sound interventions. Specifically, the soothing voice group exhibited lower activation in the dorsolateral and ventrolateral PFC, including Broca's Region. In contrast, significant differences related to the stress task were observed in the orbitofrontal PFC regions, accompanied by right lateralization, which is associated with stress responses and emotional regulation [42]. Interestingly, while the soothing voice did not directly reverse this lateralization, it shifted activation to distinct regions, including Brodmann area 45. This area is involved in semantic language processing [43] and executive functions, such as the theory of mind, and cognitive empathy [44,45], suggesting that a soothing voice may engage processes related to emotional regulation and social cognition.

### 6.3 Sympathetic responses and EDA

While EDA levels increased significantly during the stress induction phase across all groups, no significant differences were observed among groups during the recovery phase. This result suggests that autonomic recovery patterns were not differentially modulated by the sound interventions. However, the observed decrease in EDA levels during the recovery phase shows the overall effectiveness of the task protocol in both evoking and resolving sympathetic activation.

The discrepancy between significant effects in cortisol and fNIRS measures versus the lack of differences in EDA recovery may reflect the sensitivity of these biomarkers to distinct aspects of stress physiology. While cortisol and fNIRS capture central and systemic stress responses, EDA primarily reflects peripheral sympathetic activation. Such peripheral responses may be less sensitive to the more delicate influences of prosodic elements in speech, which could explain the lack of differential modulation in EDA recovery.

### 6.4 Implications and future directions

The current findings have several practical implications. First, they underscore the potential of paralanguage-based interventions in stress recovery, with applications in clinical, educational, and occupational settings. For example, voice awareness and modulation training could be beneficial in helping professions and mental health care, particularly for individuals in high-stress environments or those recovering from traumatic experiences.

Future research should investigate how individual differences, such as baseline stress reactivity or cultural background, may influence responses to paralanguage interventions. In addition, integrating other neuroimaging modalities, such as EEG or MRI, could provide a more comprehensive understanding of the neural circuits involved in paralanguage processing and stress modulation.

### 6.5 Limitations

Several limitations of this study should be acknowledged. The relatively small sample size may limit the generalizability of the findings. Furthermore, the study design relied on a single standardized audio track, which may not capture the variability in individual preferences for voice qualities. Finally, the acute nature of the stress induction and recovery phases limits our ability to assess the long-term efficacy of voice interventions for stress management.

## 7 Conclusion

This study provides multimodal evidence that paralinguistic elements of human speech can accelerate physiological stress recovery. Our results partially confirmed the study hypothesis. Specifically, we observed a significantly faster decline in salivary cortisol levels in the soothing voice group compared to both robotic voice and silence conditions, supporting the hypothesis that non-semantic vocal elements facilitate recovery on the biochemical level. In contrast, electrodermal activity did not show differential recovery across groups, suggesting that peripheral sympathetic markers may be less sensitive to prosodic modulation in the absence of semantic or visual engagement.

Hemodynamic data from fNIRS further supported the hypothesis by revealing that the soothing voice condition induced distinct patterns of prefrontal activation and lateralization. These changes included reduced dorsolateral and ventrolateral prefrontal activity, implying a neurophysiological mechanism through which paralanguage may promote stress resolution.

Taken together, these findings suggest that paralanguage independent of speech content holds therapeutic potential as a non-invasive, voice-based tool for stress recovery. This study highlights the importance of acoustic prosody in shaping physiological stress outcomes and provides a neurobiological foundation for the development of future auditory interventions in mental health care, human–AI communication, and stress-sensitive environments.

## CRediT authorship contribution statement

**Marina Saskovets:** Writing – review & editing, Writing – original draft, Visualization, Validation, Project administration, Methodology, Formal analysis, Data curation, Conceptualization. **Mykhailo Lohachov:** Writing – review & editing, Visualization, Software, Data curation. **Zilu Liang:** Writing – review & editing, Supervision, Resources, Funding acquisition.

## Financial disclosure

The current study was funded by the regular laboratory budget of the Ubiquitous and Personal Computing Lab, Kyoto University of Advanced Science. The first author, Marina Saskovets, was supported by the Tamai Scholarship during the completion of this work. The second author, Mykhailo Lohachov, received support from the Japan Science and Technology Agency SPRING, Grant Number JPMJSP2180.

## Declaration of competing interest

The authors declare that they have no known competing financial interests or personal relationships that could have appeared to influence the work reported in this paper.

## Abbreviations

ASMR: Autonomous Sensory Meridian Response
EDA: Electrodermal Activity
EDF: European Data Format
EEG: Electroencephalography
MRI: Magnetic Resonance Imaging
fNIRS: Functional Near-Infrared Spectroscopy
HPA: Hypothalamic–Pituitary–Adrenal (axis)
ICS: Immunochromatographic Strip
LIR: Laterality Index Response
nM: Nanomolar
OHb/ΔOHb: Oxygenated Hemoglobin/Change in Oxygenated Hemoglobin
HHb/ΔHHb: Deoxygenated Hemoglobin/Change in Deoxygenated Hemoglobin
PFC: Prefrontal Cortex
ROI: Region of Interest
SCI: Scalp Coupling Index
SCL: Skin Conductance Level
SD: Standard Deviation
Tx/Rx: Transmitter/Receiver (fNIRS optodes)
